# Supplementary material for: Predicting visual function by interpreting a neuronal wiring diagram
Source: Nature. 2024 Oct 2;634(8032):113–23. doi: 10.1038/s41586-024-07953-5 (PMC11446822; doi:10.1038/s41586-024-07953-5)
Supplement: Supplementary file 6 — CRF and ERF predictions for individual TmY4 and TmY9 cells. Analogous to Supplementary Data 3, but for TmY target types. Shown are the top four monosynaptic pathways, the strongest pathway passing through each of the top ten intermediary types (ranking from Extended Data Fig. 7), and the trisynaptic pathway Tm1–TmY–Dm3–TmY (see the section entitled Prediction of spatial normalization). [file 41586_2024_7953_MOESM6_ESM.zip › DataS4/TmY9q/720575940626624328.pdf]

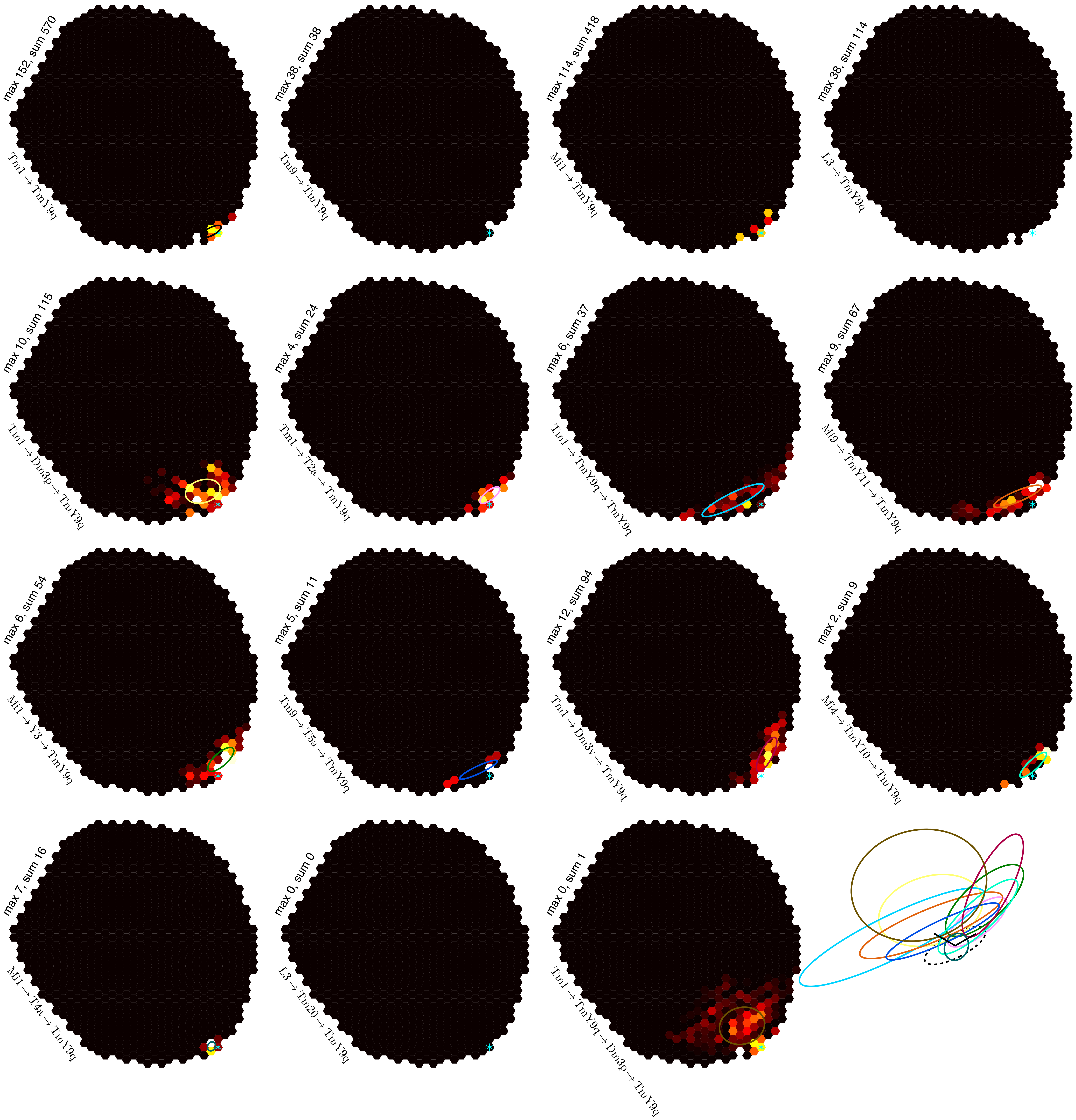

max 152, sum 570
Tm1 → TmY9q
max 38, sum 38
Tm9 → TmY9q
max 114, sum 418
Mi1 → TmY9q
max 38, sum 114
L3 → TmY9q
max 10, sum 115
Tm1 → Dm3p → TmY9q
max 4, sum 24
Tm1 → T2a → TmY9q
max 6, sum 37
Tm1 → TmY9q → TmY9q
max 9, sum 67
Mi9 → TmY11 → TmY9q
max 6, sum 54
Mi1 → Y3 → TmY9q
max 5, sum 11
Tm9 → T5a → TmY9q
max 12, sum 94
Tm1 → Dm3v → TmY9q
max 2, sum 9
Mi4 → TmY10 → TmY9q
max 7, sum 16
Mi1 → T4a → TmY9q
max 0, sum 0
L3 → Tm20 → TmY9q
max 0, sum 1
Tm1 → TmY9q → Dm3p → TmY9q
